# Context-Dependent Association Between Serum 25-Hydroxyvitamin D and Romosozumab Bone Mineral Density Response: A Stratified Analysis by Renal Function Category and Prior Treatment History in a Real-World Japanese Cohort

**DOI:** 10.3390/nu18101642

**Published:** 2026-05-21

**Authors:** Ryo Nakano, Ayumi Ichisawa, Kenya Saruta, Masakazu Kogawa, Akira Fukuda

**Affiliations:** Department of Orthopedic Surgery, Mutsu General Hospital (Shimokita Medical Center Association), 1-2-8 Ogawamachi, Mutsu 035-8601, Aomori, Japan; mohumohu31@gmail.com (A.I.); saruken82@yahoo.co.jp (K.S.); masakazu_k_0512@yahoo.co.jp (M.K.); asafukuda@k5.dion.ne.jp (A.F.)

**Keywords:** romosozumab, 25-hydroxyvitamin D, bone mineral density, renal function, TRACP-5b, prior treatment history, osteoporosis, Japan

## Abstract

**Background/Objectives:** Serum 25-hydroxyvitamin D (25OHD) is a key determinant of calcium-phosphorus homeostasis and bone metabolism; however, its role as a modifier of the bone mineral density (BMD) response to romosozumab, a dual-action anabolic agent for osteoporosis, remains poorly characterized, particularly across different levels of renal function. This study investigated whether the renal function category and prior treatment history modified the association between baseline 25OHD and romosozumab BMD response. **Methods:** We conducted a retrospective cohort study of 315 consecutive Japanese patients treated with romosozumab (210 mg subcutaneously monthly for 12 months) at Mutsu General Hospital, Aomori, Japan (2019–2025; IRB approval RO7-5; date of approval: 20 January 2026). Patients were stratified by eGFR-based renal function category: preserved renal function (eGFR ≥ 60 mL/min/1.73 m^2^, n = 199) and moderately reduced renal function (eGFR 30–59 mL/min/1.73 m^2^, n = 86). Patients with severely reduced renal function (eGFR 15–29, n = 11) were excluded from the comparative analyses. Spearman rank correlations (Rs) were computed between baseline 25OHD and (i) baseline TRACP-5b and (ii) 12-month lumbar spine BMD changes. Mediation analysis was performed to examine TRACP-5b as a potential mediator. **Results:** In the preserved renal function group, baseline 25OHD was significantly and inversely correlated with TRACP-5b (Rs = −0.246, *p* = 0.0007). This correlation was absent in the moderately reduced renal function group (Rs = +0.036, *p* = 0.74), and the interaction was statistically significant (z = −2.38, *p* = 0.017). Among treatment-experienced patients, lower 25OHD levels were correlated with greater LS-BMD response (Rs = −0.197, *p* = 0.036), whereas no such correlation was observed in treatment-naïve patients (Rs = −0.009, *p* = 0.902). Mediation analysis did not identify TRACP-5b as a significant mediator. **Conclusions:** The association between serum 25OHD and romosozumab BMD response appears to be context-dependent across renal function categories and prior treatment history. These findings are hypothesis-generating and require prospective validation before they can be applied to clinical practice.

## 1. Introduction

Romosozumab, a humanized monoclonal antibody targeting sclerostin, exerts a unique dual mechanism of simultaneously increasing bone formation and decreasing bone resorption, producing rapid and substantial gains in bone mineral density (BMD) in postmenopausal women with osteoporosis [1,2]. The FRAME and ARCH trials demonstrated 73% and 48% reductions in vertebral fracture risk, respectively, establishing romosozumab as the most potent anabolic agent currently available [3,4]. Despite this efficacy, the magnitude of the BMD response varies considerably among patients in real-world clinical practice [5,6,7].

Vitamin D status, as measured by serum 25-hydroxyvitamin D (25OHD), is a critical determinant of bone metabolism [8]. In vitamin D-sufficient individuals, adequate 25OHD ensures efficient intestinal calcium absorption and suppresses parathyroid hormone (PTH)-driven bone resorption [9]. In states of vitamin D deficiency, elevated PTH stimulates osteoclast activity through RANKL upregulation, elevating bone turnover markers, including tartrate-resistant acid phosphatase 5b (TRACP-5b), which is a recognized predictor of romosozumab BMD response [10].

Renal function is an important modifier of vitamin D metabolism. As eGFR declines, fibroblast growth factor 23 (FGF-23) levels rise early in the course of renal function decline and directly suppress renal CYP27B1 (1alpha-hydroxylase) activity while stimulating CYP24A1 (24-hydroxylase), thereby reducing calcitriol synthesis [11],12. Importantly, FGF-23 elevation has been detected before substantial reductions in eGFR and before significant increases in PTH or phosphate levels in patients with early renal function impairment [12]. Therefore, the relationship between serum 25OHD and bone resorption may be attenuated in patients with moderately reduced renal function.

A history of osteoporosis treatment was an additional modifier. Antiresorptive agents profoundly suppress TRACP-5b levels and are well-established negative predictors of romosozumab BMD response [13,14]. Whether 25OHD levels differentially predict BMD response across renal function categories and treatment history subgroups remains unknown.

We conducted this retrospective cohort study to examine whether the relationship between baseline serum 25OHD and romosozumab BMD response is context-dependent and modified by (i) renal function category and (ii) prior treatment history.

## 2. Materials and Methods

### 2.1. Study Design and Participants

This retrospective cohort study reviewed the medical records of all patients who initiated romosozumab treatment between April 2019 and April 2025 at the Mutsu General Hospital (Shimokita Medical Center Association), Aomori Prefecture, Japan. The inclusion criteria were as follows: (1) diagnosis of osteoporosis [15]; (2) completion of 12 monthly romosozumab injections (210 mg subcutaneously); and (3) availability of baseline serum 25OHD, TRACP-5b, and BMD measurements at 12 months. The final cohort comprised 315 patients. The STROBE checklist is provided in Supplementary Table S1.

This study was approved by the Institutional Review Board of Mutsu General Hospital (approval number: RO7-5; date of approval: 20 January 2026) and was conducted in accordance with the Declaration of Helsinki. The requirement for informed consent was waived using an opt-out approach.

Companion manuscript disclosure: This manuscript is part of a series of independent analyses from the same prospective registry cohort (n = 315; IRB RO7-5). A companion manuscript examining vitamin D formulation type as a predictor of romosozumab BMD response has been published [16]. There was no overlap in the primary outcomes, statistical models, or conclusions between the two manuscripts.

### 2.2. Data Collection

Baseline data included age, sex, BMI, prior osteoporosis treatment history, current vitamin D co-administration, and comorbidities, including diabetes mellitus and underlying renal disease etiology. The laboratory parameters included serum 25OHD (ECLIA; Elecsys Vitamin D Total II, Roche Diagnostics, Basel, Switzerland), TRACP-5b (ELISA; Nittobo Medical, Tokyo, Japan), P1NP (ECLIA; Elecsys Total P1NP, Roche Diagnostics, Basel, Switzerland), eGFR (mL/min/1.73 m^2^), albumin-corrected calcium, and iPTH (Elecsys PTH, Roche Diagnostics, Basel, Switzerland). All blood samples were collected in the morning after overnight fasting.

### 2.3. Outcome Measurements and Renal Function Stratification

BMD was measured using DXA (Hologic Discovery) at the lumbar spine (L1-L4; LS-BMD) and total hip (TH-BMD) at baseline and 12 months. The primary outcome was the percentage change in LS-BMD from baseline to 12 months. The eGFR was calculated using the equation provided by the Japanese Society of Nephrology [17].

Patients were stratified into eGFR-based renal function categories: preserved renal function (eGFR > 60 mL/min/1.73 m^2^, n = 199), moderately reduced renal function (eGFR 30–59 mL/min/1.73 m^2^, n = 86), and severely reduced renal function (eGFR 15–29 mL/min/1.73 m^2^, n = 11). These categories were based on eGFR alone. Formal KDIGO-based chronic kidney disease (CKD) staging additionally requires markers of kidney damage, such as albuminuria or proteinuria, which were not available in our dataset. Therefore, the renal function categories used throughout this manuscript should not be interpreted as KDIGO-defined CKD stages; some patients in the eGFR ≥ 60 category may not have CKD, and some with eGFR 30–59 may have conditions other than CKD. This limitation is further discussed in Section 5.

Patients with severely reduced renal function (eGFR 15–29, n = 11) were excluded from all comparative and correlation analyses owing to insufficient statistical power and qualitatively different clinical management. All primary analyses included 285 patients.

### 2.4. Statistical Analysis

The Shapiro–Wilk test was applied to all continuous variables. Normally distributed variables (age, BMI, LS-BMD, TH-BMD, eGFR, corrected calcium, and 12-month LS-BMD change) are presented as mean ± SD; non-normally distributed variables (25OHD, TRACP-5b, P1NP, and iPTH) are presented as median (IQR). Between-group comparisons were performed using the Student’s *t*-test or the Mann–Whitney *U* test, as appropriate. Categorical variables were compared using the chi-square or Fisher’s exact test.

The primary analysis used Spearman rank correlation (Rs) to examine the relationships between baseline 25OHD and (i) baseline TRACP-5b and (ii) 12-month LS-BMD change, computed separately within each renal function stratum and treatment history stratum. Prespecified primary analyses examined the preserved versus moderately reduced renal function and treatment-naïve versus treatment-experienced comparisons. Interaction tests were performed using the method described by Altman and Bland [18]. A post hoc power calculation for the treatment-history interaction (Rs = −0.009 versus Rs = −0.197) using the method of [19] estimated the power at approximately 52%, indicating a substantial risk of type II error. No formal correction for multiple comparisons was applied, and secondary analyses should be interpreted with caution. The sensitivity analysis using multivariable regression with formal interaction terms is presented in Supplementary Table S4; a sensitivity analysis adjusting for active vitamin D analogue co-administration is presented in Supplementary Table S2.

Mediation analysis used the Baron–Kenny causal steps framework [20]. Disclosure of generative AI assistance: Claude (Anthropic) was used for language editing and structure. Figures were generated using Python (matplotlib 3.8.4, scipy 1.13.0); no AI image generation tool was used. All authors take full responsibility for the study’s content.

## 3. Results

### 3.1. Baseline Characteristics

The baseline characteristics stratified by renal function category are presented in Table 1 (n = 285 for the primary analysis). The overall cohort (n = 315) was predominantly female (96.8%) and had a mean age of 77.0 ± 8.1 years, with profound vitamin D deficiency (median 25OHD 13.8 ng/mL; 89.2% below 30 ng/mL). The moderately reduced renal function group was significantly older and had higher iPTH levels. The prevalence of diabetes mellitus was 6.0% versus 8.1% (*p* = 0.52). The proportion of treatment-experienced patients was 40.9% (*p* = 0.37, between strata).

### 3.2. 25OHD-TRACP-5b Correlation Stratified by Renal Function Category

**Figure 1:**
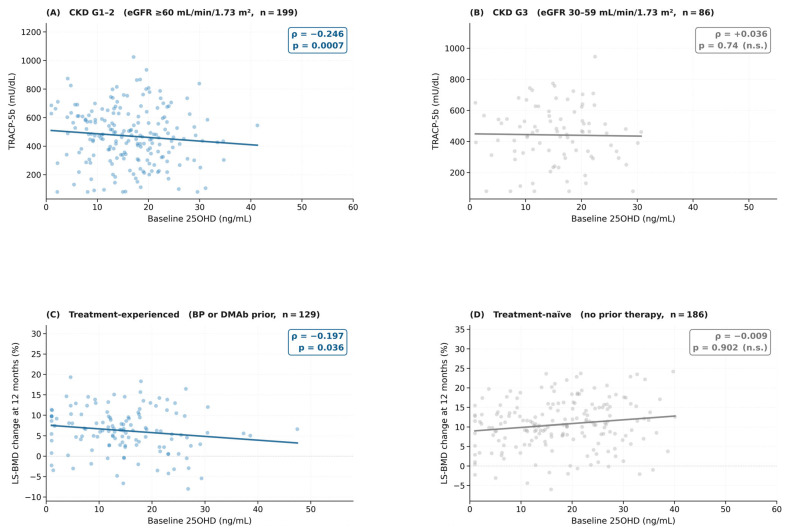
Scatter plots showing context-dependent Spearman rank correlations between baseline serum 25-hydroxyvitamin D (25OHD) and bone metabolism outcomes (n = 285). (**A**) Preserved renal function (eGFR ≥ 60 mL/min/1.73 m^2^, n = 199): significant inverse correlation between 25OHD and TRACP-5b (Rs = −0.246, *p* = 0.0007). (**B**) Moderately reduced renal function (eGFR 30–59 mL/min/1.73 m^2^, n = 86): no significant correlation (Rs = +0.036, *p* = 0.74). (**C**) Treatment-experienced patients (n = 129): significant inverse correlation between 25OHD and 12-month LS-BMD change (Rs = −0.197, *p* = 0.036). (**D**) Treatment-naïve patients (n = 186): no significant correlation (Rs = −0.009, *p* = 0.902). Solid lines represent Theil-Sen monotone regression lines fitted to the data for visual reference only; Blue lines and dots indicate statistically significant correlations (*p* < 0.05); grey lines and dots indicate non-significant correlations (*p* ≥ 0.05).these lines illustrate the direction of Spearman’s rank correlation and do not constitute inferential regression models. No confidence or prediction bands are shown in the figure. 25OHD, 25-hydroxyvitamin D; TRACP-5b, tartrate-resistant acid phosphatase 5b; BMD, bone mineral density; Rs, Spearman’s rank correlation coefficient; n.s., not significant.

The Spearman rank correlation between baseline 25OHD and TRACP-5b was significantly negative in the preserved renal function group (eGFR ≥ 60, n = 199): Rs = −0.246, *p* = 0.0007 (Table 2, Figure 1A). This correlation was absent in the moderately reduced renal function group (eGFR 30–59, n = 86): Rs = +0.036, *p* = 0.74 (Figure 1B). The interaction test was statistically significant (z = −2.38; *p* = 0.017). The overall cohort correlation was not significant (Rs = −0.078, *p* = 0.168).

### 3.3. 25OHD-BMD Response Correlation Stratified by Prior Treatment History

Among treatment-experienced patients (n = 129), lower 25OHD levels were significantly correlated with greater LS-BMD response (Rs = −0.197, *p* = 0.036; Table 3, Figure 1C). No such correlation was observed in treatment-naïve patients (Rs = −0.009, *p* = 0.902; Figure 1D). The interaction test was borderline (z = −1.73, *p* = 0.084), and post hoc power was approximately 52%, indicating a substantial type II error risk. Within the treatment-experienced subgroup, the lowest 25OHD quartile showed a mean LS-BMD gain of 8.2 ± 6.1% versus 5.1 ± 4.8% in the highest quartile (difference: 3.1 percentage points).

### 3.4. Mediation Analysis

Path a (25OHD to TRACP-5b; beta = −3.21, *p* = 0.003) and path b (TRACP-5b to LS-BMD change; beta = 0.012, *p* = 0.001) were both significant. However, the indirect effect in the treatment-experienced subgroup was not significant (beta = −0.038, 95% bootstrap CI −0.098 to +0.015, *p* = 0.17). These findings are hypothesis-generating.

### 3.5. BMD Response Outcomes

Overall, LS-BMD increased by 8.90 ± 6.50% and TH-BMD by 2.90 ± 3.80% at 12 months (Table 4). LS-BMD gains did not differ significantly between the preserved and moderately reduced renal function groups (9.2 ± 6.6% versus 8.3 ± 6.1%, *p* = 0.27). Treatment-naïve patients showed significantly greater LS-BMD gains (10.9 ± 6.4% versus 6.5 ± 5.8%, *p* < 0.001).

## 4. Discussion

This study suggests that the association between serum 25OHD and romosozumab BMD response may be context-dependent, varying by eGFR-based renal function category and prior treatment history of osteoporosis. All findings should be interpreted as hypothesis-generating, given the retrospective design, eGFR-only renal function classification, modest correlation magnitudes, and statistical limitations described below.

### 4.1. Renal Function-Modified 25OHD-TRACP-5b Correlation

The absence of an inverse 25OHD-TRACP-5b correlation in the moderately reduced renal function group (eGFR 30–59) is consistent with impaired vitamin D bioactivation at lower eGFR levels. As eGFR declines, renal CYP27B1 (1alpha-hydroxylase) activity may be progressively impaired, reducing the conversion of 25OHD to calcitriol and thereby attenuating the downstream suppressive effects of vitamin D on bone resorption [11,21].

However, it is important to note that CKD-related disruption of vitamin D metabolism is not dichotomous at the eGFR threshold used in this study. FGF-23, secreted by osteocytes in response to phosphate retention, directly suppresses CYP27B1 activity and stimulates CYP24A1 (24-hydroxylase), thereby reducing the synthesis of calcitriol. Importantly, FGF-23 levels are elevated before substantial declines in eGFR and before significant increases in PTH or serum phosphate levels in patients with early renal function impairment [12]. Therefore, some degree of vitamin D bioactivation impairment may already be present in patients with preserved eGFR (≥60), and conversely, the extent of impairment in patients with eGFR 30–59 is likely heterogeneous. The eGFR-based stratification used in this study is a practical approximation of a biological continuum rather than a precise biological threshold, and the mechanistic interpretation should be understood in this context. These findings are consistent with current recommendations to use active vitamin D analogs rather than native vitamin D supplementation in patients with substantially reduced renal function [22,23], but do not constitute evidence that vitamin D repletion should be deprioritized.

### 4.2. Treatment-History-Modified 25OHD-BMD Response Correlation

The inverse correlation between 25OHD and BMD response in treatment-experienced patients requires careful interpretation. Regression to the mean and confounding by prior antiresorptive suppression severity are plausible alternative explanations for this finding. The inverse correlation (Rs = −0.197, *p* = 0.036) should be considered as hypothesis-generating. The 3.1 percentage point difference in LS-BMD gain between the lowest and highest 25OHD quartiles in treatment-experienced patients (8.2% versus 5.1%) may not translate to a clinically meaningful reduction in fracture risk, and direct extrapolation would be speculative.

### 4.3. Mediation and Clinical Implications

The non-significant TRACP-5b mediation suggests that TRACP-5b-independent pathways may be involved. Our findings do not recommend vitamin D repletion in any patient group; vitamin D adequacy remains a universal therapeutic goal consistent with current guidelines [22,23,24,25]. This study tentatively suggests that serum 25OHD may have limited predictive utility as a biomarker for romosozumab BMD response in patients with moderately reduced renal function or without prior antiresorptive therapy [26], pending prospective validation [27]. A companion study from the same cohort examining vitamin D formulation type as a predictor of romosozumab response will be reported separately [16].

## 5. Limitations

This study had several important limitations. First, the retrospective, single-center design precluded causal inference. Second, serum 25OHD was measured at a single time point without systematic recording of the month of sampling, precluding seasonal adjustments. Third, additional unmeasured confounders included dietary calcium intake, sunlight exposure, and adherence to supplementation. Fourth, active vitamin D metabolites (calcitriol) were not measured; in patients with reduced renal function, 25OHD levels may overestimate vitamin D bioactivity [28]. Fifth, the study population was predominantly women (96.8%). Sixth, no formal correction for multiple comparisons was performed. Seventh, the interaction tests were underpowered (estimated power of approximately 52%). Eighth, regression to the mean may have contributed to the observed inverse correlation in treatment-experienced patients. Ninth, the mediation analysis used the Baron–Kenny framework; modern counterfactual methods would provide more robust estimates. Finally, extrapolation outside Japan requires independent validation. Eleventh, and critically, albuminuria and proteinuria data were not available in our dataset, precluding formal KDIGO-based CKD staging in our study. The renal function categories used throughout this manuscript are defined by eGFR thresholds alone and may include patients without CKD as well as patients with undiagnosed CKD in the preserved renal function group. This limitation directly affects the generalizability of the mechanistic interpretation and further underscores the hypothesis-generating nature of the stratified analyses.

## 6. Conclusions

The association between serum 25OHD and romosozumab BMD response appears context-dependent across eGFR-based renal function categories and prior treatment history, although these findings are hypothesis-generating and require prospective validation. In patients with preserved renal function (eGFR ≥ 60), 25OHD may suppress bone resorptive activity via TRACP-5b; this potential relationship may be attenuated in patients with moderately reduced renal function (eGFR 30–59), consistent with the progressive impairment of vitamin D bioactivation. However, the absence of albuminuria data limits the strength of the mechanistic inference, and the eGFR threshold used represents a practical approximation of a biological continuum involving FGF-23-mediated and 1alpha-hydroxylase-mediated pathways. These findings do not advocate vitamin D repletion, which remains a standard therapeutic goal. Prospective studies with formal CKD staging, calcitriol measurement, and FGF-23 profiling are required.

## Figures and Tables

**Table 1:** Baseline characteristics stratified by renal function category (n = 285).

Variable	Preserved RF (eGFR ≥ 60, n = 199)	Mod. Reduced RF (eGFR 30–59, n = 86)	*p* Value
Age, years	76.1 +/− 7.9	79.3 +/− 8.5	0.002
Female sex, n (%)	193 (97.0)	82 (95.3)	0.51
BMI, kg/m^2^	21.6 +/− 3.4	20.9 +/− 3.1	0.08
LS-BMD (%YAM)	69.8 +/− 13.2	67.4 +/− 12.8	0.13
TH-BMD (%YAM)	58.3 +/− 11.4	55.9 +/− 10.7	0.09
25OHD, ng/mL †	14.2 (9.8–20.1)	12.8 (8.9–18.4)	0.14
TRACP-5b, mU/dL †	463 (298–641)	451 (291–619)	0.60
P1NP, microg/L †	68.4 (38.2–98.6)	65.1 (36.8–93.2)	0.71
eGFR, mL/min/1.73 m^2^	75.2 +/− 11.3	44.1 +/− 8.2	<0.001
iPTH, pg/mL †	36.2 (24.8–51.4)	50.1 (34.2–71.3)	<0.001
Corrected Ca, mg/dL	9.01 +/− 0.38	8.97 +/− 0.41	0.40
Renal disease etiology, n (%)			
Hypertensive nephropathy	76 (38.2)	38 (44.2)	0.34
Diabetic nephropathy ‡	24 (12.1)	16 (18.6)	0.14
Chronic glomerulonephritis	39 (19.6)	13 (15.1)	0.37
Unknown/other	60 (30.1)	19 (22.1)	0.17
Diabetes mellitus, n (%) §	12 (6.0)	7 (8.1)	0.52
Prior treatment, n (%)	80 (40.2)	36 (41.9)	0.37
Active VitD use, n (%)	78 (39.2)	38 (44.2)	0.41

Data are presented as mean ± SD, median (IQR), or n (%). RF, renal function category (eGFR-based; not the KDIGO-defined CKD stage). † indicates variables that were non-normally distributed (Shapiro–Wilk *p* < 0.05) ‡ Diabetic nephropathy (renal disease etiology subrow) specifically refers to renal disease attributed to diabetes. § Diabetes mellitus (comorbidity row) indicates the overall prevalence of diabetes as a systemic comorbidity, regardless of renal disease etiology. Patients with diabetic nephropathy (‡) are included in both rows; there is no double counting between other etiology categories. BMI, body mass index; LS-BMD, lumbar spine BMD; TH-BMD, total hip BMD; 25OHD, 25-hydroxyvitamin D; TRACP-5b, tartrate-resistant acid phosphatase 5b; P1NP, procollagen type I *N*-terminal propeptide; eGFR, estimated glomerular filtration rate; iPTH, intact parathyroid hormone; Ca, calcium; VitD, vitamin D; %YAM, percentage of young adult mean.

**Table 2:** Spearman rank correlations between baseline 25OHD and TRACP-5b levels by renal function category (n = 285).

Renal Function Category	n	Spearman Rs	*p* Value	Interpretation
Preserved RF (eGFR ≥ 60)	199	−0.246	0.0007 *	Significant inverse correlation
Mod. Reduced RF (eGFR 30–59)	86	+0.036	0.74	No significant correlation
Overall (n = 285)	285	−0.078	0.168	No significant correlation

* *p* < 0.05. Interaction test: z = −2.38, *p* = 0.017; RF, renal function category (eGFR-based). Rs, Spearman’s rank correlation coefficient. 25OHD: 25-hydroxyvitamin D; TRACP-5b: tartrate-resistant acid phosphatase 5b.

**Table 3:** Spearman rank correlations between baseline 25OHD and 12-month LS-BMD changes (%) by treatment history.

Treatment History	n	Spearman Rs	*p* Value	Interpretation
Treatment-naive	186	−0.009	0.902	No significant correlation
Treatment-experienced	129	−0.197	0.036 *	Significant inverse correlation
Overall	315	−0.078	0.168	No significant correlation

* *p* < 0.05. Interaction test naive vs. experienced: z = −1.73, *p* = 0.084 (post hoc power approx. 52%). Rs, Spearman’s rank correlation coefficient.

**Table 4:** BMD response outcomes according to renal function category and prior treatment history.

Subgroup	n	LS-BMD Change (%)	TH-BMD Change (%)	*p* Value †
Overall cohort (n = 315)	315	8.90 +/− 6.50	2.90 +/− 3.80	—
Preserved RF (eGFR ≥ 60)	199	9.2 +/− 6.6	3.1 +/− 3.9	Ref
Mod. Reduced RF (eGFR 30–59)	86	8.3 +/− 6.1	2.6 +/− 3.5	0.27
Treatment-naive	186	10.9 +/− 6.4	3.5 +/− 3.9	Ref
Treatment-experienced	129	6.5 +/− 5.8	2.0 +/− 3.5	<0.001

† *p*-value versus reference (Student’s *t*-test). RF, renal function category. The severely reduced RF group (eGFR 15–29, n = 11) was excluded (see Supplementary Table S3).

## Data Availability

Data are available upon request from the corresponding author (IRB RO7-5).

## Supplementary Materials

The following supporting information can be downloaded at: https://www.mdpi.com/article/10.3390/nu18101642/s1. Table S1: STROBE Checklist for Observational Studies (Cohort Study); Table S2: Sensitivity Analysis: Spearman Rank Correlations Stratified by Active Vitamin D Analogue Co-administration; Table S3: Descriptive Statistics for the Severely Reduced Renal Function Group (eGFR 15–29 mL/min/1.73 m^2^, n = 11); Table S4: Sensitivity Analysis: Multivariable Linear Regression with Formal Interaction Terms.
