# Supplementary material for: Context-Dependent Association Between Serum 25-Hydroxyvitamin D and Romosozumab Bone Mineral Density Response: A Stratified Analysis by Renal Function Category and Prior Treatment History in a Real-World Japanese Cohort
Source: Nutrients. 2026 May 21;18(10):1642. doi: 10.3390/nu18101642 (PMC13209380; doi:10.3390/nu18101642)
Supplement: Supplementary file 1 [file nutrients-18-01642-s001.zip › nutrients-4292415-supplementary.pdf]

Supplementary Materials

Manuscript ID: nutrients-4292415

Context-Dependent Association Between Serum 25-Hydroxyvitamin D and Romosozumab Bone Mineral Density Response: A Stratified Analysis by Renal Function Category and Prior Treatment History in a Real-World Japanese Cohort

Table S1. STROBE Checklist for Observational Studies (Cohort Study)

Item	Topic	Recommendation	Page No.	Reported
	**Title and Abstract**			
1	Title and abstract	(a) Indicate the study's design with a commonly used term in the title or the abstract (b) Provide in the abstract an informative and balanced summary of what was done and what was found	1	Yes — Title states 'retrospective cohort study'; Abstract provides structured summary
	**Introduction**			
2	Background/rationale	Explain the scientific background and rationale for the investigation being reported	1–2	Yes — Section 1 (Introduction)
3	Objectives	State specific objectives, including any pre-specified hypotheses	2	Yes — Section 1, final paragraph
	**Methods**			
4	Study design	Present key elements of study design early in the paper	2	Yes — Section 2.1
5	Setting	Describe the setting, locations, and relevant dates, including periods of recruitment, exposure, follow-up, and data collection	2	Yes — Mutsu General Hospital, April 2019–April 2025
6	Participants	(a) Give the eligibility criteria, and the sources and methods of selection of participants (b) Describe methods of follow-up	2	Yes — Section 2.1: inclusion/exclusion criteria described
7	Variables	Clearly define all outcomes, exposures, predictors, potential confounders, and effect modifiers. Give diagnostic criteria, if applicable	2–3	Yes — Sections 2.2 and 2.3
8	Data sources/measurement	For each variable of interest, give sources of data and details of methods of assessment (measurement). Describe comparability of assessment methods if there is more than one group	2–3	Yes — Section 2.2: assay methods specified

9	Bias	Describe any efforts to address potential sources of bias	3, 8–9	Yes — Sections 2.4 and 5 (Limitations)
10	Study size	Explain how the study size was arrived at	2	Yes — consecutive enrolment; n=315 stated
11	Quantitative variables	Explain how quantitative variables were handled in the analyses. If applicable, describe which groupings were chosen and why	3	Yes — Section 2.4: Shapiro-Wilk, continuous vs. categorical
12	Statistical methods	(a) Describe all statistical methods, including those used to control for confounding (b) Describe any methods used to examine subgroups and interactions (c) Explain how missing data were addressed (d) Report any sensitivity analyses	3–4	Yes — Section 2.4
	**Results**			
13	Participants	(a) Report numbers of individuals at each stage of study (b) Consider use of a flow diagram	4	Yes — Figure/Section 3.1; n=315 overall, n=285 primary analysis
14	Descriptive data	(a) Give characteristics of study participants (b) Indicate number of participants with missing data for each variable of interest	4–5	Yes — Table 1
15	Outcome data	Report numbers of outcome events or summary measures	5–7	Yes — Tables 2–4
16	Main results	(a) Give unadjusted estimates and, if applicable, confounder-adjusted estimates and their precision (b) Report category boundaries when continuous variables were categorized (c) If relevant, consider translating estimates of relative risk into absolute risk for a meaningful time period	5–7	Yes — Tables 2–4, Figure 1
17	Other analyses	Report other analyses done—e.g., analyses of subgroups and interactions, and sensitivity analyses	6–7	Yes — Sections 3.3, 3.4; Supplementary Table S4
	**Discussion**			
18	Key results	Summarise key results with reference to study objectives	7	Yes — Section 4, opening paragraph
19	Limitations	Discuss limitations of the study, taking into account sources of potential bias or imprecision. Discuss both direction and magnitude of any potential bias	8–9	Yes — Section 5 (11 limitations enumerated)

20	Interpretation	Give a cautious overall interpretation of results considering objectives, limitations, multiplicity of analyses, results from similar studies, and other relevant evidence	7–9	Yes — Sections 4 and 6
21	Generalisability	Discuss the generalisability (external validity) of the study results	9	Yes — Section 5, limitation 10
	**Other Information**			
22	Funding	Give the source of funding and the role of the funders for the present study and, if applicable, for the original study on which the present article is based	—	Yes — Funding section: no external funding

STROBE = Strengthening the Reporting of Observational Studies in Epidemiology. This checklist is based on the STROBE statement for cohort studies (von Elm et al., Lancet 2007; 370:1453-1457).

Table S2. Sensitivity Analysis: Spearman Rank Correlations Stratified by Active Vitamin D Analogue Co-administration

To address potential confounding by active vitamin D analogue (VDA; eldecalcitol or alfacalcidol) co-administration, primary correlations were re-examined after stratifying by VDA use. Active VDA was prescribed in 116/315 patients (36.8%) overall.

Comparison	n	Spearman Rs	p value	Interpretation
**25OHD vs. TRACP-5b — Preserved RF (eGFR >=60)**				
With VDA co-administration (n = 78)	78	-0.229	0.044 *	Significant inverse correlation
Without VDA co-administration (n = 121)	121	-0.258	0.004 *	Significant inverse correlation
**25OHD vs. TRACP-5b — Moderately Reduced RF (eGFR 30-59)**				
With VDA co-administration (n = 38)	38	+0.051	0.76	No significant correlation
Without VDA co-administration (n = 48)	48	+0.021	0.88	No significant correlation
**25OHD vs. LS-BMD change — Treatment-Experienced**				
With VDA co-administration (n = 52)	52	-0.188	0.18	No significant correlation
Without VDA co-administration (n = 77)	77	-0.201	0.080	Borderline correlation
**25OHD vs. LS-BMD change — Treatment-Naive**				
With VDA co-administration (n = 64)	64	-0.014	0.91	No significant correlation
Without VDA co-administration (n = 122)	122	-0.005	0.96	No significant correlation

** $p < 0.05$. Rs, Spearman rank correlation coefficient. RF, renal function category. VDA, active vitamin D analogue (eldecalcitol or alfacalcidol). LS-BMD, lumbar spine bone mineral density. Results are consistent with the primary analysis: the inverse correlation between 25OHD and TRACP-5b in the preserved RF group persists irrespective of VDA co-administration, confirming that this finding is not attributable to VDA use.*

Table S3. Descriptive Statistics for the Severely Reduced Renal Function Group (eGFR 15-29 mL/min/1.73 m², n = 11)

This subgroup was excluded from all comparative and primary correlation analyses due to insufficient statistical power and qualitatively different clinical management (mandatory active vitamin D analogues, intensified phosphate monitoring). Descriptive statistics are provided here for completeness.

Variable	Severely Reduced RF (eGFR 15-29, n = 11)
Age, years	80.2 ± 9.1
Female sex, n (%)	11 (100.0)
BMI, kg/m2	20.1 ± 2.8
LS-BMD (%YAM)	64.1 ± 11.4
TH-BMD (%YAM)	52.3 ± 9.8
eGFR, mL/min/1.73 m2	22.4 ± 4.1
25OHD, ng/mL †	10.8 (7.2-15.6)
TRACP-5b, mU/dL †	491 (318-674)
P1NP, microg/L †	72.1 (41.3-108.4)
iPTH, pg/mL †	88.4 (52.1-124.3)
Corrected Ca, mg/dL	8.81 ± 0.52
Prior treatment, n (%)	5 (45.5)
Active VitD use, n (%)	11 (100.0)
12-month LS-BMD change (%)	7.8 ± 5.9
12-month TH-BMD change (%)	2.2 ± 3.1

Data are mean ± SD or median (IQR) †, or n (%). † Non-normally distributed variables (Shapiro-Wilk $p < 0.05$). Note: All 11 patients in this group received active vitamin D analogues (eldecalcitol or alfacalcidol) as part of standard CKD-MBD management. 25OHD, 25-hydroxyvitamin D; TRACP-5b, tartrate-resistant acid phosphatase 5b; P1NP, procollagen type I N-terminal propeptide; eGFR, estimated glomerular filtration rate; iPTH, intact parathyroid hormone; BMI, body mass index; VitD, vitamin D; %YAM, percentage of young adult mean.

Table S4. Sensitivity Analysis: Multivariable Linear Regression with Formal Interaction Terms

Multivariable linear regression models were fitted with 25OHD as the primary predictor of 12-month LS-BMD change (%), with formal interaction terms for (Model A) 25OHD × renal function category (preserved vs. moderately reduced) and (Model B) 25OHD × prior treatment history (naive vs. experienced). All models were adjusted for age (years), baseline LS-BMD (%YAM), and eGFR (mL/min/1.73 m²) as continuous covariates. These analyses complement the primary Spearman rank correlation analyses and serve as a sensitivity check.

Model A: 25OHD × Renal Function Category Interaction (n = 285)

Variable	Beta	95% CI	p value	Interpretation
25OHD (ng/mL)	-0.08	-0.22 to +0.06	0.26	Non-significant main effect
Renal function category (ref: preserved)	-0.41	-1.84 to +1.02	0.57	Non-significant main effect
25OHD × RF category (interaction term)	+0.31	+0.04 to +0.58	0.024 *	Significant interaction
Age (years)	-0.09	-0.21 to +0.03	0.14	—
Baseline LS-BMD (%YAM)	-0.18	-0.31 to -0.05	0.006 *	—
eGFR (mL/min/1.73 m2)	+0.02	-0.04 to +0.08	0.52	—

Model B: 25OHD × Prior Treatment History Interaction (n = 315)

Variable	Beta	95% CI	p value	Interpretation
25OHD (ng/mL)	-0.04	-0.14 to +0.06	0.43	Non-significant main effect
Prior treatment (ref: naive)	-3.82	-5.11 to -2.53	<0.001 *	Significant main effect
25OHD × Treatment history (interaction term)	-0.19	-0.41 to +0.03	0.091	Borderline interaction
Age (years)	-0.07	-0.18 to +0.04	0.21	—
Baseline LS-BMD (%YAM)	-0.16	-0.27 to -0.05	0.004 *	—
eGFR (mL/min/1.73 m2)	+0.01	-0.04 to +0.06	0.71	—

** $p < 0.05$. Beta coefficients represent the change in 12-month LS-BMD change (%) per unit increase in the predictor variable. CI, confidence interval. RF, renal function category (0 = preserved, eGFR ≥60; 1 = moderately reduced, eGFR 30–59). Prior treatment (0 = naive; 1 = experienced). Model A: The significant 25OHD × RF category interaction ($p = 0.024$) indicates that the association between 25OHD and LS-BMD change differs significantly by renal function category, consistent with the primary Spearman analysis. Model B: The borderline 25OHD × treatment history interaction ($p = 0.091$) is consistent with the borderline result in the primary analysis (interaction test $p = 0.084$). LS-BMD, lumbar spine bone mineral density; eGFR, estimated glomerular filtration rate.*
